# Prevalence of urogenital, anal, and pharyngeal infections with *Chlamydia trachomatis*, *Neisseria gonorrhoeae*, and *Mycoplasma genitalium*: a cross-sectional study in Reunion island

**DOI:** 10.1186/s12879-021-05801-9

**Published:** 2021-01-21

**Authors:** A. Calas, N. Zemali, G. Camuset, J. Jaubert, R. Manaquin, C. Saint-Pastou, Y. Koumar, P. Poubeau, P. Gerardin, A. Bertolotti

**Affiliations:** 1 CHU Réunion, Service des Maladies Infectieuses – Dermatologie, Saint Pierre, La Réunion France; 2 CHU Réunion, Laboratoire de microbiologie, Saint Pierre, La Réunion France; 3 Inserm CIC1410, CHU Réunion, Saint Pierre, La Réunion France

**Keywords:** Sexually transmitted infections, Screening, *Chlamydia trachomatis*, *Neisseria gonorrhoeae*, *Mycoplasma genitalium*, Reunion island

## Abstract

**Background:**

Recommendations for sexually transmitted infection (STI) screening vary significantly across countries. This study evaluated the prevalence of urogenital and extragenital infections with *Chlamydia trachomatis* (CT), *Neisseria gonorrhoeae* (NG), and *Mycoplasma genitalium* (MG) in patients visiting a French STI clinic in the Indian Ocean region to determine whether current STI screening practices should be updated.

**Methods:**

This cross-sectional study examined all patients who visited the STI clinic between 2014 and 2015. Triplex polymerase chain reaction screening for CT, NG, and MG was performed on urine, vaginal, pharyngeal, and anal specimens (FTD Urethritis Basic Kit, Fast Track Diagnostics, Luxembourg).

**Results:**

Of the 851 patients enrolled in the study, 367 were women (367/851, 43.2%) and 484 were men (484/851, 56.0%). Overall, 826 urogenital specimens (826/851, 97.1%), 606 pharyngeal specimens (606/851, 71.2%), and 127 anal specimens (127/851, 14.9%) were taken from enrolled patients. The prevalence of urogenital CT and MG was high in women ≤25 years (19/186, 10.21%; 5/186, 2.69%) and in men who have sex with women ≤30 years (16/212, 7.54%; 5/212, 2.36%). Among patients with urogenital CT infection, 13.7% (7/51) had urethritis. All patients with urogenital MG infection were asymptomatic. Men who have sex with men had a high prevalence of pharyngeal CT (2/45, 4.44%) and NG (3/44, 6.81%) and a high prevalence of anal CT (2/27, 7.41%), NG (2/27, 7.40%), and MG (1/27, 3.70%). After excluding patients with concomitant urogenital infection, extragenital infections with at least 1 of the 3 pathogens were found in 20 swabs (20/91, 21.9%) taken from 16 patients (16/81, 19.7%), all of them asymptomatic.

**Conclusions:**

Routine multisite screening for CT, NG, and MG should be performed to mitigate the transmission of STIs in high-risk sexually active populations.

**Supplementary Information:**

The online version contains supplementary material available at 10.1186/s12879-021-05801-9.

## Background

Sexually transmitted infections (STIs) are a major public health concern, especially since their prevalence is increasing globally [1]. When left untreated, infections with *Chlamydia trachomatis* (CT), *Neisseria gonorrhoeae* (NG), and *Mycoplasma genitalium* (MG) can cause severe long-term complications, including pelvic inflammatory disease, ectopic pregnancy, infertility, chronic pelvic pain, and neurological or cardiovascular diseases [2]. Because infected patients are often asymptomatic, screening for these pathogens presents a major challenge. International STI guidelines [3] strongly encourage annual screening for urogenital CT and NG in men who have sex with men (MSM), sexually active women ≤25 years, at-risk women > 25 years, and at-risk men who have sex with women (MSW) (risky behaviour being defined here as having new or multiple sex partners, having received a recent STI diagnosis, having sex for money, or having sex while on drugs). Although routine screening for extragenital CT and NG in MSM and at-risk women (defined as women who engage in anal or oral sex) is recommended in some developed countries (i.e. France, Canada, and Australia), STI guidelines published in other countries, both developed and developing, do not make specific recommendations for extragenital infections. Lastly, available recommendations regarding MG infection generally discourage the screening of asymptomatic patients regardless of infection site to prevent the overprescription of azithromycin and the resulting increase in antimicrobial resistance. This is perplexing considering that MG infection is the second most common cause of nongonococcal urethritis [4].

As extragenital infections can occur without concomitant urogenital infection [5], symptom-based urogenital screening alone may cause a large number of STIs to go undetected and untreated. Moreover, this screening approach can generate a false sense of security in asymptomatic patients, which in turn can facilitate the spread of STIs in the population. This is a major issue in low- and middle-income regions like Reunion Island, as these are often home to high-risk sexually active populations.

This study evaluated the prevalence of urogenital, anal, and pharyngeal infections with CT, NG, and MG in patients visiting an STI clinic in Reunion Island to determine whether STI screening practices should be updated.

## Methods

This monocentric cross-sectional study examined all patients who visited a major STI clinic in Reunion Island between June 2014 and August 2015. All patients were asked to complete an anonymous questionnaire that collected data on age, marital status, level of education, drug use, sexual activity, urogenital symptoms, and extragenital (i.e. pharyngeal or anal) symptoms (Additional file 1). Urine specimens were collected from men, vaginal self-swab specimens from women, and pharyngeal and anal swab specimens from at-risk patients (i.e. men and women who engage in anal and/or oral sex) and MSW volunteers. All specimens were screened by triplex polymerase chain reaction (PCR) for CT, NG, and MG (FTD Urethritis Basic Kit, Fast Track Diagnostics, Luxembourg). Specimens with cycle threshold values ≤38 and exponential amplification curves were considered positive. Patients also underwent serological tests for HIV, HBV, and HCV. Quantitative variables were expressed as means with standard deviation (SD), and prevalence and qualitative variables were expressed as percentages. The 95% confidence intervals (95% CI) of STI prevalence estimates were calculated using adjusted binomial distribution. Statistical analyses were performed using R software (R Core Team 2019, R Foundation for Statistical Computing, Vienna, Austria). A *P*-value < 0.05 was considered statistically significant.

Oral informed consent was obtained from all participants and written informed consent was obtained from a parent or guardian for participants under 18 years old. The ethical character of oral consent alone for this study on totally anonymous collected data was approved by the Scientific Committee for research of the CHU Réunion. No review by an ethical committee was required (article R1121–1, decree n°2017–884 of 9 May 2018 - art.2). The study was registered with the National Institute of Health Data (MR 0912200220) in accordance with French regulations and was conducted using the MR-004 reference methodology of the Commission Nationale Informatique et Libertés (CNIL).

## Results

**Fig. 1:**
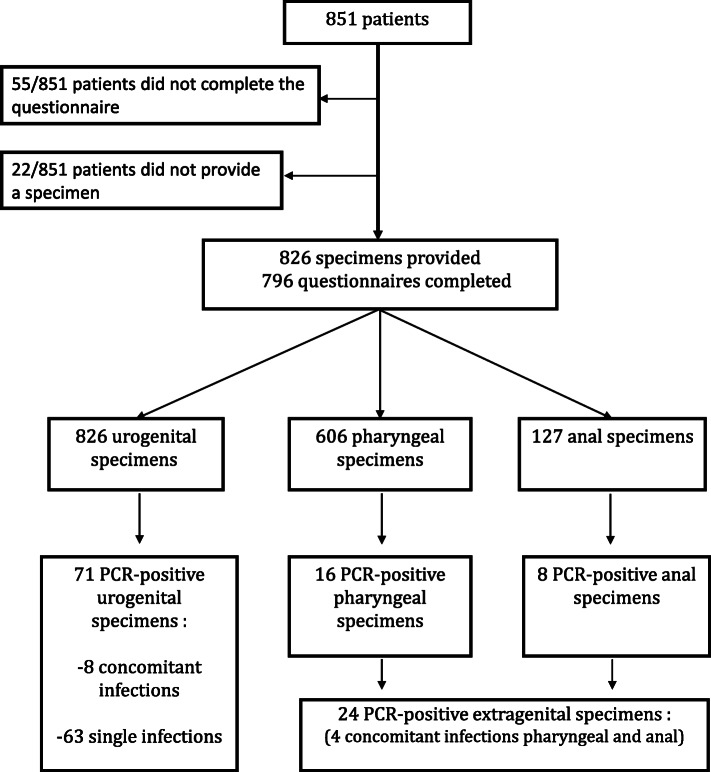
Flow chart of enrolled patients

Over the 14-month period, 851 patients were enrolled in the study, including 367 women (367/851, 43.2%) and 484 men (484/851, 56.0%). Of these, 796 patients (796/851, 95.5%) completed the questionnaire; questionnaires were not obtained from the other 55 patients (55/851, 6.5%) because they were wrongly administered. No significant difference was observed in STI prevalence between respondents and non-respondents. A total of 53 men (53/484, 10.9%) were MSM. Mean age was 28.95 (14–70, SD 10.61) years. Overall, 826 urogenital specimens (826/851, 97.1%), 606 pharyngeal specimens (606/851, 71.2%), and 127 anal specimens (127/851, 14.9%) were taken from the 851 enrolled patients (Fig. 1). A total of 95 specimens (95/1559, 6.1%) taken from 85 patients (85/851, 9.9%, mean age 26.31 years, 15–70, SD 9.84) were positive for at least 1 of the 3 pathogens, including 71 urogenital specimens (71/826, 8.6%), 16 pharyngeal specimens (16/606, 2.6%), and 8 anal specimens (8/127, 6.2%). Table 1 shows the prevalence of CT, NG, and MG in the 3 types of specimen. Three CT-MG co-infections and 7 CT-NG co-infections were found. Pharyngeal and anal infections with concomitant urogenital infection were excluded from the analysis. The overall prevalence of CT in urogenital, pharyngeal, and anal specimens was 6.17% (51/826), 1.98% (12/606), and 7.87% (10/127), respectively. The prevalence of NG in urogenital, pharyngeal, and anal specimens was 0.73% (6/826), 1.48% (9/606), and 3.15% (4/127), respectively. The prevalence of MG in urogenital, pharyngeal, and anal specimens was 1.81% (15/826), 0.33% (2/606), and 2.36% (3/127) respectively.

**Table 1:** Prevalence of *Chlamydia trachomatis*, *Neisseria gonorrhea, Mycoplasma genitalium* in the 3 types of specimen (Reunion Island, 2014–2015)

	Urogenital specimen			Pharyngeal specimen^a^			Anal specimen^a^		
	n/N	*P*-value^e^	Prevalence (95%CI^d^)	n/N	*P*-value^e^	Prevalence (95CI%^d^)	n/N	*P*-value^e^	Prevalence (95CI%^d^)
*Chlamydia trachomatis* infection									
Women ≤25y	19/186^b^	**0.02**	**10.21 (6.26–15.49)**	4/141^f^	0.12	**2.83 (0.78–7.10)**	2/36^f,h^	0.7	**5.56 (0.68–18.66)**
Women >25y	7/159^b^	–	4.40 (1.79–8.86)	0/134	–	0.00 (0.00–2.72)	0/49	–	0.00 (0.00–7.26)
MSM	2/53		3.77 (0.46–12.98)	2/45^f^		**4.44 (0.54–15.15)**	2/27^f,h^		**7.41 (0.91–24.29)**
MSW ≤30y	16/212	**0.05**	**7.54 (4.37–11.97)**	1/147	0.63	0.68 (0.02–3.73)	0/2		0.00 (0.00–84.19)
MSW >30y	4/124	–	3.23 (0.89–8.05)	0/88	–	0.00 (0.00–4.11)	0/1		0.00 (0.00–97.50)
Other men^c^	2/92		2.17 (0.26–7.63)	1/47		**2.13 (0.05–11.29)**	0/8		0.00 (0.00–36.94)
*Neisseria gonorrhea* infection									
Women ≤25y	2/186	0.41	1.07 (0.13–3.83)	0/139	0.5	0.00 (0.02–2.62)	0/38		0.00 (0.00–9.25)
Women >25y	1/159	–	0.63 (0.01–3.45)	0/133		0.00 (0.00–2.74)	0/50		0.00 (0.00–7.11)
MSM	2/53		**3.77 (0.46–12.98)**	3/44^g^		**6.81 (1.43–18.66)**	2/27^g^		**7.40 (0.91–24.29)**
MSW ≤30y	1/212	0.63	0.47 (0.01–2.60)	1/147	0.26	0.68 (0.02–3.73)	0/2		0.00 (0.00–84.19)
MSW >30y	0/124	–	0.00 (0.00–2.93)	2/88	–	**2.27 (0.28–7.97)**	0/1		0.00 (0.00–97.50)
Other men^c^	0/92		0.00 (0.00–3.97)	0/47		0.00 (0.00–7.55)	0/8		0.00 (0.00–36.94)
*Mycoplasma genitalium* infection									
Women ≤25y	5/186	0.2	**2.69 (0.88–6.16)**	1/145	0.5	0.72 (0.02–4.09)	0/39	0.56	0.00 (0.07–9.03)
Women >25y	2/159	–	1.26 (0.15–4.47)	1/134	–	0.75 (0.02–4.12)	1/50	–	**2.00 (0.05–10.65)**
MSM	1/53		1.89 (0.05–10.07)	0/45		0.00 (0.00–7.87)	1/27		**3.70 (0.09–18.97)**
MSW ≤30y	5/212	0.1	**2.36 (0.77–5.42)**	0/147		0.00 (0.00–2.48)	0/2		0.00 (0.00–84.19)
MSW >30y	0/124	–	0.00 (0.00–2.93)	0/88		0.00 (0.00–4.11)	0/1		0.00 (0.00–97.50)
Other men^c^	2/92		2.17 (0.26–7.63)	0/47		0.00 (0.00–7.55)	0/8		0.00 (0.00–36.94)

^a^Prevalence of anal and pharyngeal infections without concomitant urogenital CT, NG, or MG infection; *y* years; ^b^Total excludes one woman with CT infection for whom age was unknown; *MSM* men who have sex with men, *MSW* men who have sex with women; ^c^men who did not share their sexual orientation; *CI* confidence interval; ^d^binomial exact confidence interval; ^e^Fisher’s exact test; n: number of positive specimens; *N* total number of specimens; ^f^Total includes one specimen with concomitant pharyngeal and anal infections; ^g^Total includes two patients with concomitant pharyngeal and anal infections; ^h^no anorectitis reported by infected patients

The prevalence of urogenital CT infection was 10.21% (19/186, 95%CI 6.26–15.49%, *p =* 0.02) in women ≤25 years and 7.54% (16/212, 95% CI 4.37–11.97%, *p =* 0.05) in MSM.

The prevalence of anal CT infection without concomitant urogenital infection was 5.56% (2/36 95%CI 0.68–18.66%, *p =* 0.7) in women ≤25 years and 7.41% (2/27, 95%CI 0.91–24.29%) in MSM. The prevalence of pharyngeal CT infection without concomitant urogenital infection was 2.83% (4/141, 95%CI 0.78–7.10%, *p =* 0.12) in women ≤25 years and 4.44% (2/45, 95%CI 0.54–15.15%) in MSM.

The prevalence of anal NG infection without concomitant urogenital infection was 7.40% (2/27, 95%CI 0.91–24.29%) in MSM. The prevalence of pharyngeal NG infection without concomitant urogenital infection was 6.81% (3/44, 95%CI 1.43–18.66%) in MSM.

The prevalence of anal MG infection without concomitant urogenital infection was 2% (1/50, 95%CI 0.05, 10.65%) in women > 25 years and 3.70% (1/27, 95%CI 0.09–18.97%) in MSM. The prevalence of pharyngeal MG infection without concomitant urogenital infection was 0.72% (1/145, 95%CI 0.02–4.09%, *p =* 0.5) in women ≤25 years and 0.75% (1/134, 95%CI 0.02–4.12%) in women > 25 years.

After excluding patients with concomitant urogenital infection and MSW volunteers, extragenital infections with at least 1 of the 3 pathogens were found in 20 swabs (20/91, 21.9%) taken from 16 patients (16/81, 19.7%), all of them asymptomatic. Among patients with urogenital CT infection, 13.7% (7/51 had urethritis. Among patients with urogenital NG infection, 16.6% (1/6) had hematuria and 16.6% (1/6) had urethritis. All patients with urogenital MG infection were asymptomatic.

## Discussion

This study evaluated the prevalence of urogenital, anal, and pharyngeal infections with CT, NG, and MG in patients visiting an STI clinic in Reunion Island to determine whether current STI screening practices should be updated. A high prevalence of urogenital CT and MG was found in both women ≤25 years (19/186, 10.21%; 2.69%) and MSW ≤30 years (16/212, 7.54%; 2.36%). Moreover, MSM were found to have a high prevalence of pharyngeal CT (2/45, 4.44%) and NG (3/44, 6.81%) and a high prevalence of anal CT (2/27, 7.41%), NG (2/27, 7.40%), and MG (1/27, 3.70%). These figures are comparable to those reported for Africa, but are higher than those reported for Europe and South East Asia [1].

Three MSW volunteers were tested positive for pharyngeal infection with NG. As this infection is unlikely to occur in low-risk patients, we concluded that our PCR technique produced false positives due to cross reaction of NG and *Neisseria meningitidis*. Accordingly, we excluded all MSW volunteers from our calculations of extragenital infection prevalence. Thus, extragenital infections with at least 1 of the 3 pathogens were found in 20 swabs (20/91, 21.9%) taken from 16 patients (16/81, 19.7%), all of them asymptomatic.

The high prevalence of extragenital CT and NG infections suggests that many STIs are left undetected with current screening practices. This finding is supported by a cross-sectional study of 4402 women at risk of extragenital infection who visited an American STI clinic: the authors concluded that 30.3% of NG infections and 13.8% of CT infections would have been missed if only urogenital infections had been investigated [6]. Likewise, a US cross-sectional study of 7333 MSM visiting an STI clinic found that a third of pharyngeal and anal infections with NG would have been be missed if only urethral or urine specimens had been screened [7]. In turn, the high percentage of asymptomatic patients in our study (72/81, 88.9%) suggests that symptom-based urogenital screening alone can contribute to underestimating the prevalence of urogenital STIs. More generally, this screening approach can generate a false sense of security in asymptomatic patients, which can favour the progression of silent STIs towards irreversible complications [2]. This can be a major concern in low- and middle-income countries, which are often home to high-risk sexually active populations.

Screening for urogenital and extragenital MG raises its own set of issues, as the overprescription of azithromycin increases antimicrobial resistance in patients treated for this infection [8]. In Reunion Island, the prevalence of MG-CT co-infections is high (OR 4,62, *p* = 0.02) and several risk factors for CT infection have been identified, including first intercourse between ages 11 and 14 (*p* = 0.02), lack of previous screening (*p* = 0.023), and partner infidelity (*p* = 0.012). In view of these findings, our research team argued elsewhere that routine screening for MG in poorly adherent, high-risk populations living in regions with low antimicrobial resistance may help limit the spread of MG, thereby preventing irreversible complications, especially in women (Torregrossa E, Zemali N, Gerardin P, Manaquin R, Foucher A, Jaubert J, Picot S, Poubeau P, Camuset G, Bertolotti A: Prevalence, risk factors, and symptomatology of Mycoplasma genitalium infection in Reunion Island: A cross-sectional study, submitted) (Duval C, Anthony N, Thore-Dupont E, Jaubert J, Camuset G, Von Theobald P, Franco JM, Poubeau P, Bruneau L, Bertolotti A: Prevalence and risk factor of Chlamydia trachomatis infection among women consulting sexually transmitted infection centre in La Reunion: A cross-sectional study, submitted).

## Conclusion

To conclude, health care providers should be made aware of the high prevalence of extragenital CT and NG infections and urogenital and extragenital MG infections in sexually active populations. Screening practices should be updated as follows: multisite CT and NG screening should be routinely performed in patients at risk of extragenital infection, and multisite MG screening should be routinely performed in poorly-adherent, high-risk populations living in regions with low antimicrobial resistance [9]. This screening strategy may help mitigate the transmission of STIs, including in low-income countries, which are often home to high-risk populations.

## Supplementary Information

**Additional file 1.**

## Data Availability

The data used during the study are available from the corresponding author.

## Abbreviations

STI: Sexually transmitted Infection
CT: *Chlamydia trachomatis*
NG: *Neisseria gonorrhoeae*
MG: *Mycoplasma genitalium*
MSM: Men who have sex with men
MSW: Men who have sex with women
PCR: Polymerase chain reaction
SD: Standard deviation
95% CI: 95% Confidence interval
