# Supplementary material for: Prevalence of urogenital, anal, and pharyngeal infections with Chlamydia trachomatis, Neisseria gonorrhoeae, and Mycoplasma genitalium: a cross-sectional study in Reunion island
Source: BMC Infect Dis. 2021 Jan 21;21:95. doi: 10.1186/s12879-021-05801-9 (PMC7818901; doi:10.1186/s12879-021-05801-9)
Supplement: Supplementary file 1 — Additional file 1. [file 12879_2021_5801_MOESM1_ESM.pdf]

Valid ☒ Not valid ☐

Anonymization number

Do not fold

# Anonymous STI (Sexually Transmitted Infection) Questionnaire

CHU La Réunion, Saint Pierre

**Month of consultation :** ☐Jan. ☐Feb. ☐Mar. ☐April ☐May ☐June ☐July ☐Aug. ☐Sept. ☐Oct.☐Nov.☐Dec.
**Year of consultation :** ☐2014 ☐2015 ☐2016

**Sex** ☐ Male ☐ Female

**Age** *dizaine* ☐1☐2☐3☐4☐5☐6☐7☐8☐9
*unit* ☐0☐1☐2☐3☐4☐5☐6☐7☐8☐9

**You are :** ☐ Single ☐ In a relationship ☐ Married

**Reason for consultation :** ☐ Recent risk taking ☐ Clinical signs suggestive of STIs ☐ Screening because STI at partner's ☐ Health check / end of relationship ☐ Desire to stop using a condom / new relationship ☐ Control at 6 weeks ☐ Others

**Have you stayed in the last 2 years in :** ☐ Madagascar ☐ South Africa ☐ West Africa ☐ East Africa ☐ North Africa ☐ Comoros ☐ Asia ☐ Australia ☐ South America ☐ Eastern Europe

**If you have taken a recent risk :**

It was : ☐ 1 day ago ☐ 2 to 3 days ago ☐ 4 to 7 days ago ☐ 7 to 15 days ago ☐15 to 30 days ago ☐ more than 30 days
It was : ☐ A man ☐ A woman
It was in one of the countries mentioned above ☐ Yes ☐ No
It was at : ☐ la souris chaude ☐ St Gilles ☐ St Pierre ☐ Etang salé ☐ Pierrefond / Pointe du diable ☐ St Louis ☐ St Paul ☐ St Joseph ☐ Tampon ☐ Ligne Paradis ☐ Bois d'olive ☐ Ligne des Bambous ☐ Terre sainte ☐ Ravine blanche ☐ other ……………………..
It was : ☐ at the beach ☐ at your home ☐ at her/him ☐ in nightclub/bar/pub ☐ in nature ☐ at work ☐ in a hotel ☐ other……

**Have you ever been screened for sexually transmitted diseases?** ☐ Yes ☐ No
**If yes :** it was ☐ less than 1 month ☐ 1-3 months ☐ 3-6 months ☐ 6-12 months ☐ 1 to 2 years ☐ more than 2 years

**Have you ever had / Have you:** ☐ syphilis ☐ HIV ☐ hepatitis B ☐ hepatitis C ☐ gonococcus ☐ chlamydia ☐ herpes on sex ☐ I have never had these diseases

**You have :** ☐ urinary burns ☐ sex purulent flow ☐ blood while urinating
☐ sex erosions/ulcerations ☐ mouth erosions/ulcerations
☐ anus pain/burns ☐ recent pimples on the skin
☐ I have no symptom ☐ I have other symptoms

**How long have you had one of these signs:** ☐ 1 day ☐ 2 to 3 days ☐ 4 to 7 days ☐ 7 to 15 days ☐ 15 to 30 days ☐ > 30 days

**In the last 12 months, how many different sexual partners have you had?**

Male partner(s) : ☐0☐1☐2☐3☐4☐5☐6☐7☐8☐9☐>10 Female partner(s) : ☐0☐1☐2☐3☐4☐5☐6☐7☐8☐9☐>10

**In the last 12 months, have you used a condom?**

- for vaginal penetration ☐ Always ☐ Often ☐ Rarely ☐ Never ☐ Not practiced
- for insertive fellatio (your sex in her/his mouth) ☐ Always ☐ Often ☐ Rarely ☐ Never ☐ Not practiced
- for receptive fellatio (her/his sex in your mouth) ☐ Always ☐ Often ☐ Rarely ☐ Never ☐ Not practiced
  If receptive fellatio practiced : I accepted the sample in the mouth ☐ Yes ☐ No
- for insertive anal penetrations ☐ Always ☐ Often ☐ Rarely ☐ Never ☐ Not practiced
- for receptive anal penetration ☐ Always ☐ Often ☐ Rarely ☐ Never ☐ Not practiced
  If receptive penetration is practiced : I have accepted the sample in the anus ☐ Yes ☐ No

**In the last 12 months, have you given money (or drugs) in exchange for sex?** ☐ Yes ☐ No
**In the last 12 months, have you received money (or drugs) in exchange for sex?** ☐ Yes ☐ No

**In the last 12 months, have you had a stable relationship?** ☐ Yes ☐ No

**If yes**, this partner is ☐ A man ☐ A woman

His HIV/AIDS status is ☐ Negative ☐ Positive ☐ You don't know

During your last sexual intercourse with this stable partner did you use a condom(s)?

- for fellatio ☐ Yes ☐ No ☐ Not practiced
- for vaginal or anal penetration ☐ Yes ☐ No ☐ Not practiced

**In the last 12 months, have you had occasional partners?** ☐ Yes ☐ No

**If yes** this partner is ☐ A man ☐ A woman

His HIV/AIDS status is ☐ Negative ☐ Positive ☐ You don't know

Where did you meet your occasional partners? (several choices possible)

☐ acquaintance ☐ prostitution ☐ meeting in a nightclub/bar ☐ meeting via internet ☐ Other

During your last sexual intercourse with an occasionnal partner did you use a condom(s)?

- for fellatio ☐ Yes ☐ No ☐ Not practiced
- for vaginal or anal penetration ☐ Yes ☐ No ☐ Not practiced

**Have you ever used drugs (excluding alcohol and zamal) by any route?** ☐ intravenous ☐ nasal (sniff) ☐ other

**Have you ever had sexual intercourse after consuming :**

☐ Alcohol ☐ Cannabis/zamal ☐ ecstasy ☐ cocaine ☐ heroin ☐ amphetamine ☐ GHB ☐ LSD ☐ ketamine

**Did you use a condom during sex?** ☐ Yes ☐ No

**If you are a woman :** Do you have child(ren)? ☐ Yes ☐ No

Have you been pregnant in the last 12 months? ☐ Yes ☐ No

Have you ever had spontaneous abortion? ☐ Yes ☐ No

Have you ever had difficulty getting pregnant? ☐ Yes ☐ No ☐ I did not tried

**What is the age of your first sexual intercourse? :** ☐<11 years ☐ 11 to 14 years ☐ 15 to 17 years ☐ 18 to 21 years ☐ 22 to 25 years ☐ >25 years

**What is your educational level?** ☐ Brevet ☐ Baccalaureat ☐ Higher studies ☐ Others

**What is your monthly income?** ☐ <500 € ☐ 500€-1000€ ☐ 1000€-1500€ ☐1500-2000€ ☐ 2000-3000€ ☐ 3000-4000€ ☐>4000€

☐ I am a at middle school ☐ I am a at high school ☐ I am a student

**Thank you for your participation**

Do not fold
